# The miRNA-449 family mediates doxorubicin resistance in triple-negative breast cancer by regulating cell cycle factors

**DOI:** 10.1038/s41598-019-41472-y

**Published:** 2019-03-29

**Authors:** Eduardo Tormo, Sandra Ballester, Anna Adam-Artigues, Octavio Burgués, Elisa Alonso, Begoña Bermejo, Silvia Menéndez, Sandra Zazo, Juan Madoz-Gúrpide, Ana Rovira, Joan Albanell, Federico Rojo, Ana Lluch, Pilar Eroles

**Affiliations:** 1 INCLIVA Biomedical Research Institute, Valencia, Spain; 2 Oncology and Hematology Department, Hospital Clínico Universitario-CIBERONC, Valencia, Spain; 3 Pathology Department, Hospital Clínico Universitario, Valencia, Spain; 4 Cancer Research Program, IMIM (Hospital del Mar Research Institute), Barcelona, Spain; 5 Pathology Department, IIS- Fundación Jiménez Díaz- CIBERONC, Madrid, Spain; 6 Medical Oncology Department, Hospital del Mar-CIBERONC, Barcelona, Spain; 7 Universitat Pompeu Fabra, Barcelona, Spain; 8 Universidad de Valencia, Valencia, Spain; 9 COST action CA15204, Brussels, Belgium

## Abstract

The mechanisms of chemotherapy resistance in triple negative breast cancer remain unclear, and so, new molecules which might mediate this resistance could optimize treatment response. Here we analyzed the involvement of the miRNA-449 family in the response to doxorubicin. The cell viability, cell-cycle phases, and the expression of *in silico* target genes and proteins of sensitive/resistant triple negative breast cancer cell lines were evaluated in response to doxorubicin treatment and after gain/loss of miRNAs-449 function achieved by transient transfection. Triple negative breast cancer patients were selected for *ex vivo* experiments and to evaluate gene and miRNAs expression changes after treatment, as well as survival analysis by Kaplan-Meier. Doxorubicin treatment upregulated miRNAs-449 and DNA-damage responder factors *E2F1* and *E2F3* in triple negative breast cancer sensitive breast cancer cells, while expression remained unaltered in resistant ones. *In vitro* overexpression of miRNAs-449 sensitized cells to the treatment and significantly reduced the resistance to doxorubicin. These changes showed also a strong effect on cell cycle regulation. Finally, elevated levels of miRNA-449a associated significantly with better survival in chemotherapy-treated triple negative breast cancer patients. These results reveal for the first time the involvement of the miRNA-449 family in doxorubicin resistance and their predictive and prognostic value in triple negative breast cancer patients.

## Introduction

The triple-negative breast cancer (TNBC) subtype represents the less frequent (15%) phenotype of breast cancers, however, despite its low prevalence, it is now the target of intense research because of its highly aggressive metastatic nature and very poor prognosis^1–4^. This is mainly due to its lack of specific molecular targets^5^, which means that conventional chemotherapy is the main treatment used for these patients. In this respect, anthracyclines, specifically combinations of doxorubicin and taxanes, are among the most commonly used agents^5–7^. However, resistance to these drugs and toxicities such as myelosuppression, immunosuppression, and dose-cumulative cardiotoxicity^8^ are common and represents a potential obstacle to successful treatment. The mechanisms of chemosensitivity and chemoresistance to doxorubicin are still unclear, thus, the study of regulatory pathways and possible specific targets could help optimize patient responses to this treatment. In this sense, the role of microRNAs (miRNAs) in cancer regulation and treatment responses are starting to be explored. MiRNAs are a group of endogenously expressed, non-coding small RNAs that regulate gene expression post-transcriptionally through complimentary binding to the 3′ untranslated regions (UTRs) of their target mRNAs^9^ that results in degradation of the mRNA and/or inhibition of translation^10^. Accumulating evidence indicates that miRNAs have very important regulatory functions in various cellular processes including development, metabolism, proliferation, differentiation, and apoptosis. Furthermore, increasing evidence indicates that miRNAs are associated with sensitivity or resistance to chemotherapeutic drugs, such as cisplatin or 5-fluorouracil in various cancer types^11–14^. Our group previously analyzed miRNA expression profiles in triple-negative MDA-MB-231 and MDA-MB-468 and luminal MCF-7 breast cancer cell lines after doxorubicin treatment^15^. We identified that the miRNA-449 family (miRNA-449a, miRNA-449b, miRNA-449b*, and miRNA-449c) is completely deregulated in response to doxorubicin treatment in triple-negative cell lines. Several studies have related members of this miRNA family to tumor suppression^16–20^, proliferation^21,22^, chemo-sensitivity^22^, and invasion and metastasis^23,24^ in different types of cancer. Therefore, in this study we focused on understanding their involvement in regulating and mediating the mechanisms of doxorubicin action. This could help to improve TNBC treatments or to define more efficient and less toxic alternative therapies.

## Results

### Breast cancer cells viability modulation by miRNA-449 family alone or in combination with doxorubicin

**Figure 1:**
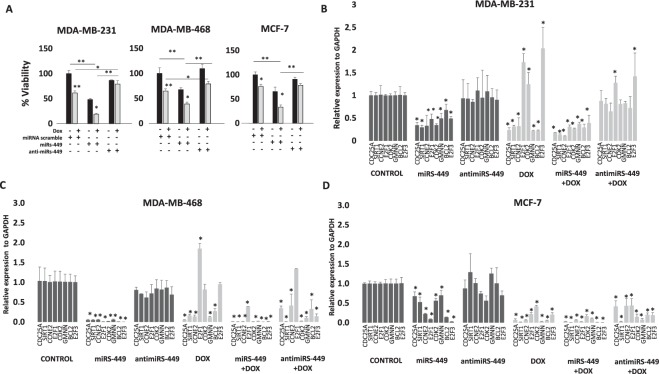
Analysis of the expression of miRNA-449 family and its target genes after transfection with miR-449s mimics or inhibitors and treatment with doxorubicin. A) Cell viability was measured by MTT after cell line transfection (MDA-MB-231, MDA-MB-468 and MCF-7) with 50 nM mimics/inhibitors of the three miRNAs (miRNA-449a, miRNA-449b and miRNA-449c) alone or in combination with 5 μM doxorubicin treatment for 24 h. Target gene expression was analysed in B) MDA-MB-231, C) MDA-MB-468, and D) MCF-7 cell lines after transfection with 50 nM mimics/inhibitors of the three miRNAs (miRNA-449a, miRNA-449b and miRNA-449c) alone or in combination with 5 μM doxorubicin for 24 h. The transfection efficiency was confirmed by qRT-PCR. Each experiment was performed in triplicate and was statistically analyzed with a Student *t*-test. Comparisons were made with respect to the transfection control of each cell line (*p < 0.05; **p < 0.01).

In a previous study of miRNAs expression profile for MDA-MB-231, MDA-MB-468, and MCF-7 breast cancer cell lines after doxorubicin treatment^15^, our group obtained miRNA-449a, miRNA-449b, and miRNA-449c overexpression in TNBC cell lines (Supplementary Fig. 1). In the present work, to investigate doxorubicin-cell susceptibility regulated by expression of these miRNAs and underlying pathways in breast cancer, experiments of gain/loss of function of this miRNA family, alone or in combination with doxorubicin treatment, were carried out. In all cases, miRNAs-449 mimics/inhibitor transfection was verified (data not shown). Doxorubicin treatment produced a viability decrease in all three cell lines: MDA-MB-231 and MDA-MB-468 viability decreased to 60% (*p* = 0.0024 and *p* = 0.0017, respectively) and MCF-7 reduced to 70% (*p* = 0.022). Gain of miRNAs-449 function (transfection with mimics) produced a statistically significant decrease in cell viability: MDA-MB-231 decreased to 50% (*p* = 0.004), and MDA-MB-468 and MCF-7 to 60% (*p* = 0.001 and *p* = 0.006, respectively). However, loss of miRNAs-449 function (transfection of inhibitors) did not produce a statistically significant difference in cell viability compared to untreated control cells (around 100%). Interestingly, combination of doxorubicin with mimics increased viability reduction compared to treatment condition alone: MDA-MB-231 decreased to 20% (*p* = 0.032), MDA-MB-468 to 40% (*p* = 0.018), and MCF-7 to 30% (*p* = 0.019). On the contrary, combination of doxorubicin with inhibitors decreased viability reduction due to the treatment: MDA-MB-231 decreased to 80% (*p* = 0.041), MDA-MB-468 to 75% (*p* = 0.038), and MCF-7 showed no differences. In summary, our results suggest a role for the miR-449 family in the modulation of cell viability when combined with doxorubicin treatment; since the mimics potentiate the effect of the drug while the inhibitors decrease it, in terms of cellular viability (Fig. 1A).

### Regulation of cell cycle and apoptosis signaling genes by miRNA-449 family

The miRNAs-449 binding sites were calculated by miRWalk v2.0 software in entire gene sequences with data from 12 different miRNAs–target interaction prediction programs. Those interactions included direct (physical) and indirect (functional) targets. Eight miRNA-449-family target genes (*CDC25A, SIRT1, GMNN, E2F1, E2F3, BCL2, CDK2*, and *CCNE2*) were selected (Supplementary Table 1); all of them involved in tumor development, cell cycle, and apoptosis (Table 1). STRING 10.5 software was used to study known and predicted protein–protein interactions between the products of different miRNA-449-family gene targets. Protein interaction predictions were derived from computational prediction, transfer of knowledge between organisms, and interactions aggregated from other databases. In our study, the strongest interaction was established between *CDK2* and *CCNE2* (score = 0. 999) (Supplementary Fig. 2). Gain/loss of miRNAs-449 function experiments were carried out in order to check their possible involvement in regulating these target genes. Doxorubicin treatment caused a decrease in expression of all the evaluated genes related to cell cycle and apoptosis, with the exception of overexpression in the *E2F1*, *E2F3*, and *CDK2* genes in MDA-MB-231, and *E2F1* in MDA-MB-468. MiRNAs-449 mimics significantly decreased the expression levels of all tested genes in all the cell lines compared to the controls, while miRNAs-449 inhibitors did not lead to significant changes in the expression of the genes analyzed. Combination of miRNAs-449 mimics and doxorubicin treatment significantly increased the downward regulatory effect of doxorubicin in *E2F1*, *E2F3*, and *CDK2*. Conversely, combination of miRNAs-449 inhibitors with doxorubicin tended to revert the effect of the drug alone, resulting in an increase in the expression of the genes evaluated. In MDA-MB-231, expression did not significantly change compared to control conditions, except for *E2F1* and *E2F3*, which showed significant overexpression (*p* = 0.03 and *p* = 0.02, respectively). However, all the target genes remained downregulated in the MDA-MB-468 and MCF7 cell lines (except for *E2F1* in MDA-MB-468 whose expression did not change compared to the control (Fig. 1B–D).

**Table 1:** Description of the selected miR-449-family target genes and their interactions.

Gene	Name	Chr	Function	miR-449 interaction	Reference
CDC25A	Cell division cycle 25A	3	Cell cycle regulation	Repressed	^20, 36^
SIRT1	Sirtuin 1	10	Regulation of cell proliferation, survival and death; plays a key role in tumorigenesis and longevity.	Repressed	^21, 22, 30^
GMNN	Geminin, DNA replication inhibitor	6	Regulation of the cell cycle: initiation of DNA replication	Repressed	^21, 31, 32^
BCL2	B-cell CLL/lymphoma 2	18	Pro-survival protein; cell protection against apoptosis.	Repressed	^22^
CDK2	Cyclin-dependent kinase 2	12	Regulation of the G1-S phase of the cell cycle; essential for the G1/S transition. This protein is associated with, and is regulated by, the regulatory subunits of cyclins E or A.	Repressed (indirectly)	^37^
E2F1	E2F transcription factor 1	20	Positive regulator of cell cycle progression. Inductor of apoptosis after DNA damage.	Feedback: Inducer Repressed (indirectly)	^22, 30, 37^
E2F3	E2F transcription factor 3	6	The E2F family plays a crucial role in cell cycle control and the action of tumor suppressor proteins.	Feedback: Inducer Repressed	^22, 30, 37^
CCNE2	Cyclin E2	8	A G1 cyclin that binds Cdk2 and plays a role in the G1/S part of the cell cycle.	Repressed	^20, 21^

### Involvement of the miRNA-449 family in doxorubicin resistance

**Figure 2:**
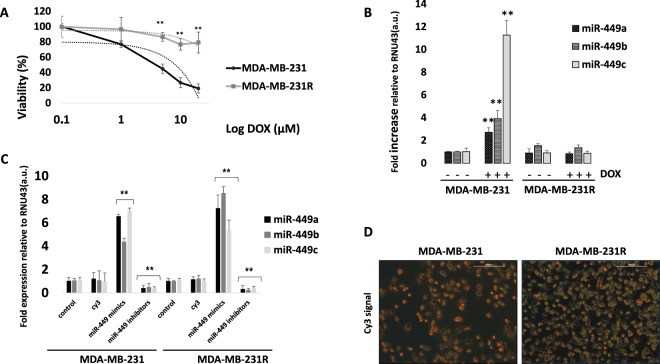
Analysis of miRNA-449 family in the MDA-MB-231 and MDA-MB-231R cell lines. (**A**) Study of cell viability in MDA-MB-231 and MDA-MB231R cell lines using MTT assays. The untreated MDA-MB-231 cells were considered to be the baseline viability control (100%) and the other conditions were compared to it. Each experiment was performed in biological and technical triplicate. The means of the results were statistically compared using a Student t-test (**p < 0.01). (**B**) Real-time PCR analysis. The ΔΔCt method was used for the analysis. The miRNA RNU43 was used as an endogenous control; expression of the miRNA-449- family miRNAs in the MDA-MB-231 was considered baseline and was statistically compared to the other conditions using a Student t-test. Each experiment was performed in biological and technical triplicate (**p < 0.01). (**C**) miRNA mimic/inhibitor transfection efficiency was measured by PCR real–time analysis in MDA-MB-231 and MDA-MB-231R cell lines. The ΔΔCt method considered control as baseline expression reference (**p < 0.01). (**D**) miRNA scramble (Cy3) transfection efficiency was measured through immunofluorescence microscopy, reaching 95% of positive signal (red staining) in MDA-MB-231 and MDA-MB-231R.

**Figure 3:**
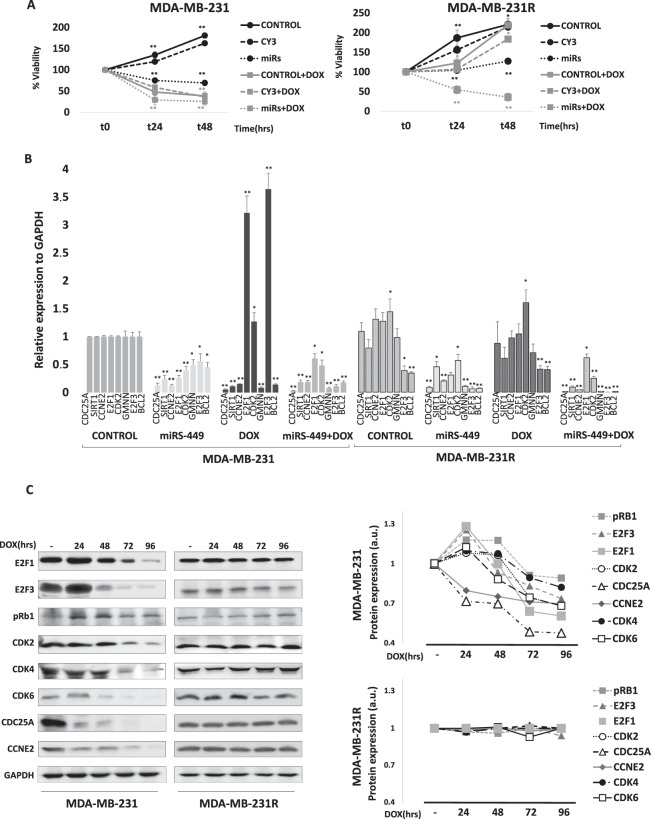
Doxorubicin response modulation by miRNA-449 family in MDA-MB-231 and MDA-MB-231R cell lines. (**A**) Cell viability study using MTT assay in MDA-MB-231 and MDA-MB-231R cells treated with doxorubicin and transfected with miRNA-449-family mimics. Untreated MDA-MB-231 was used as a viability control (100%) at time zero (t0) and the other conditions were measured in reference to it at each time point. Each experiment was performed in biological and technical triplicate. The means of the results were statistically compared using a Student t-test (*p < 0.05; **p < 0.01). CONTROL: MDA-MB-231 wild-type phenotype; CY3: MDA-MB-231 transfected with control miRNA; MiRs: MDA-MB-23/R transfected with miRNAs-449; +DOX: treatment with 5 μM doxorubicin for 24 h. (**B**) mRNA expression analysis of the miRNA-449-family gene targets in the MDA-MB-231 and MDA-MB-231R cell lines by real-time PCR after undergoing upregulation of miRs-449, doxorubicin treatment and combination of both. The ΔΔCt method was used for the analysis using *GAPDH* mRNA as the endogenous control; mRNA expression in the MDA-MB-231 cell line was considered the baseline control and was statistically compared to the other conditions using a Student *t*-test (*p < 0.05; **p < 0.01). These experiments were performed in biological and technical triplicate. (**C**) Protein levels of direct and indirect targets of miR-449 family involved in cell cycle regulation were measured by western-blotting in a doxorubicin treatment time-course (24 h, 48 h,72 h and 96 h). GAPDH was used as loading control. Graphs of signal intensity were obtained through band densitometry and referred to GAPDH and control levels.

To evaluate the possible involvement of the miRNA-449 family in doxorubicin resistance, we compared the doxorubicin-resistant MDA-MB-231R cell line with the doxorubicin-sensitive wild-type MDA-MB-231 cell line. Shift-curve viability analysis of both parental MDA-MB-231 and MDA-MB-231R with logarithmic increases of doxorubicin confirmed the MDA-MB-231R resistant phenotype (Fig. 2A). We subsequently analyzed the expression of the miRNA-449 family (miRNA-449a, miRNA-449b, and miRNA-449c) in sensitive and resistant cell lines. The experiments revealed two important results; firstly, we confirmed that miRNA-449a (~3-fold; *p* = 0.002), miRNA-449b (~4-fold; *p* = 0.001), and miRNA-449c (~11-fold; *p* = 0.00002) were overexpressed when MDA-MB-231 cells are treated with doxorubicin. Secondly, the expression of these miRNAs was not altered in MDA MB-231R after doxorubicin treatment (Fig. 2B). According with these results, both cell lines were transfected with miRNAs-449 mimics and, then, exposed to doxorubicin for 24 or 48 h. In MDA-MB-231, both the wild-type and transfection control (Cy3), cell growth progressively increased over time (~130% at 24 h and ~170% at 48 h). Conversely, the viability of cells transfected with miRNA-449 family mimics significantly decreased (~80% at 24 h, *p* = 0.003, and ~70% at 48 h, *p* = 0.0001). In the MDA-MB-231R cells, the wild-type and transfection-control cells also showed increased time-dependent growth (~180% at 24 h and ~220% at 48 h), but transfection with miRNA-449 family decreased cell viability compared with the controls (~100% at 24 h and ~120% at 48 h; Fig. 3A). Doxorubicin exposure decreased MDA-MB-231 cell viability (~50% at 24 h and 40% at 48 h), which was further decreased when combined with miRNAs-449 mimics (~30% at 24 h and 48 h; *p* = 0.001 and *p* = 0.0001, respectively). Doxorubicin treatment did not significantly change MDA-MB-231R viability, however in combination with miRNAs-449 mimics, cell viability was reduced to baseline sensitivity (~50% at 24 h and 40% at 48 h, with *p* = 0.0002 and *p* = 0.0001, respectively; Fig. 3A). These results suggest that miRNA-449 family could sensitize the resistant phenotype of MDA-MB-231R to doxorubicin, in line with the observed in Fig. 1A, in which the inhibition of this family could in part revert sensitivity of the parental MDA- MB-231 to the drug. Transfection efficiency was validated trough Real Time PCR miRNA expression (for miRNA mimics/inhibitors) and immunofluorescence staining (scramble miRNA Cy3) (Fig. 2C,D).

### Targets regulated by the miRNA-449-family in MDA-MB-231 and MDA-MB-231R cell lines

The expression of miRNA-449-family target genes was analyzed both in parental MDA-MB-231 and MDA-MB-231R cell lines treated with doxorubicin and transfected with the miRNAs. Mimic transfection resulted in significant downregulation of all the genes analyzed in MDA-MB-231, suggesting the interaction between the evaluated miRNAs and their targets. When treated with doxorubicin, these genes once again became inhibited, except for *CDK2* (~1.4-fold; *p* = 0.02), *E2F3* (~3.5-fold; *p* = 0.001), and *E2F1* (~3-fold; *p* = 0.008) which were significantly overexpressed in this cell line. Finally, combined doxorubicin and mimic treatment resulted in a return to significant reduction of all the targets. Basal levels of *E2F3* and *BCL2* were down-modulated in MDA-MB-231R compared to MDA-MB-231 cells (~0.4-fold; *p* = 0.03 and *p* = 0.01, respectively) while *CDK2* was significantly overexpressed (~1.5 fold; *p* = 0.02). As in the MDA-MB-231 cells, the transfection of MDA-MB-231R cells with mimics produced significant repression of all the evaluated targets. We also noticed that, in contrast to observations in the sensitive cell line, there was no significant change in gene expression by doxorubicin treatment. When we combined doxorubicin with miRNAs-449 mimics, all the target genes returned to dramatic reduction (Fig. 3B). Those results were also confirmed at protein level. A time- course doxorubicin treatment was carried out in order to pinpoint the main targets that mediate doxorubicin response. E2F1, E2F3, and CDK2, as well as CDK4 and CDK6 and pRB1, became initially overexpressed after doxorubicin treatment. In following time-points of treatment, expression was reduced in time dependent manner. Meanwhile, CDC25A and CCNE2 became under-expressed at the first-time point of treatment (24 h) in MDA-MB-231S cell line. On the contrary, no expression changes were appreciated after time-course doxorubicin treatment in the resistant cell line (Fig. 3C).

### Role of miRNA-449 family in cell cycle and apoptosis regulation alone or in combination with doxorubicin treatment

**Figure 4:**
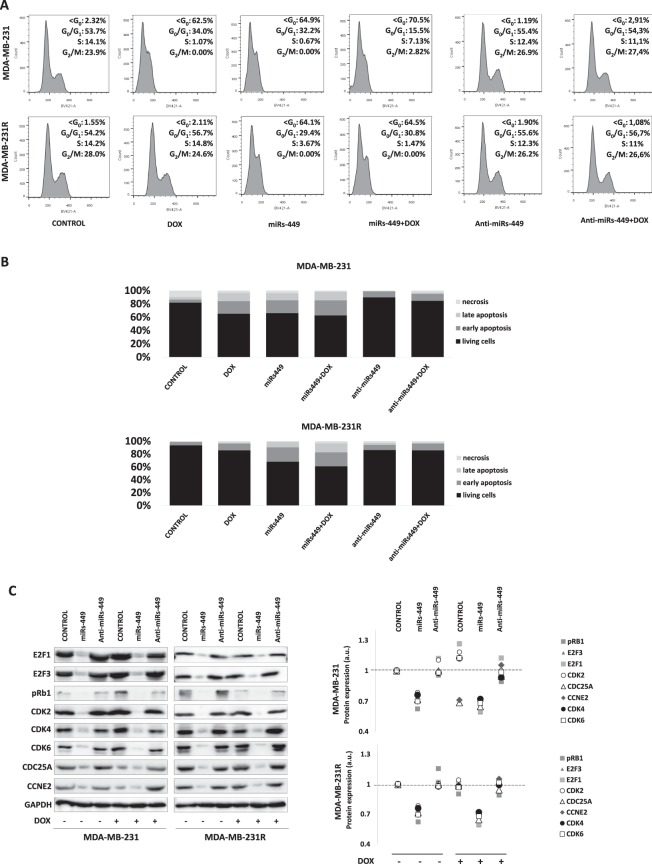
Role of miR-449 family in cell cycle and apoptosis regulation. (**A**) Flow cytometry cell-cycle study in MDA-MB-231 and MDA-MB-231R treated with doxorubicin and transfected with miRNA-449-family mimics/inhibitors. Cell-cycle histograms obtained after different treatment and transfection conditions: Y-axis, number of events (count); X axis, DAPI(BV-421A). Percentage of each cell cycle phase are included. (**B**) Apoptosis analysis obtained after treatment and transfection conditions described in (**A**); percentages of living cells, early apoptosis, late apoptosis, and necrosis are described after flow cytometry analysis. (**C**) Western blot analysis of direct and indirect targets of miR-449 family involved in cell cycle regulation. Transfection of mimics (miRs449), inhibitors (anti-miRs449), and doxorubicin treatment (DOX) conditions were considered. Graphs of signal intensity were obtained through band densitometry and referred to GAPDH and control levels.

The different behavior of sensitive versus resistant cell lines in response to doxorubicin treatment was also validated by flow cytometry cell-cycle analysis. The results confirmed the resistant phenotype of MDA-MB-231R, since the population percentages in the cell cycle phases after treatment remained similar to the control condition, in contrast to the MDA-MB-231 line, where a marked increase in the <G_0_ phase population (2.32 to 62.5%) was observed (Fig. 4A). MiRNAs-449 overexpression showed an increase of <G_0_ phase population both in sensitive and resistant cell lines, alone (2.32 to 64.9% for MDA-MB-231 and 1.55 to 64.1% for MDA-MB-231R) or in combination with doxorubicin treatment (62.5 to 70.5% for MDA-MB-231 and 2.11 to 64.5% for MDA-MB-231R). On the contrary, transfection with miRNAs-449 inhibitors did not modify the population percentages in the cell cycle phases neither in the sensitive nor in the resistant cell line, with respect to its control condition. Interestingly, miRNAs-449 inhibitors combined with the doxorubicin treatment restored the < G_0_ phase population to normal values in MDA-MB-231(62.5 to 2.91%), and continued to be similar to in MDA-MB-231R (1.08 to 2.11%) (Fig. 4A).

Apoptosis analysis results were concordant with those obtained in the cell cycle, since treatment with doxorubicin decreased the levels of living cells (81.9 to 65.3%) and increased early (4.8 to 18.8%) and late apoptosis (3.8 to 12.4%) in the MDA-MB-231, while in the MDA-MB-231R line those changes were quite lower (93.7 to 87% of living cells, 5.2 to 10% early apoptosis, and 0.1 to 1.1% late apoptosis). MiRNAs-449 mimic transfection in sensitive cell line; alone and in combination with doxorubicin treatment, produced a decrease in percentage of living cells, (81.9 to 66.1% alone; 65.3 to 62.6% combined), as well as an increase of early and late apoptosis (4.7 to 19.4% alone; 18.8 to 22.7% combined). Similar changes were also observed in resistant cell line regarding to decrease in percentage of living cells (93.7 to 68.2% alone; 85.9 to 61.1% combined) and increase of early (5.2 to 22.3% alone; 10.6 to 21.7% combined) and late apoptosis (0.1 to 8.3% alone; 1.1 to 14% combined). According also with cell cycle previous results, miRNAs-449 inhibitors transfection alone showed similar levels of living cells and early/late apoptosis in both in sensitive and resistant cell lines as their respective controls. When combined with doxorubicin treatment, percentage of living cells decreased (84.6 to 65.3%), and early (10.5 to 18.8%) and late apoptosis (1.9 to 12.4%) increased in MDA-MB-231, while no significant changes were observed in MDA-MB-231R in respect to its control (Fig. 4B).

Changes observed in cell cycle and apoptosis were also analyzed at protein level. Several proteins from genes that are directly or indirectly regulated by miRNA-449 family were blotted in same conditions as mentioned above. After doxorubicin treatment pRb1 became overexpressed in MDA-MB-231 but not in MDA-MB-231R, as well CDK4. On the contrary, CDC25A and CCNE2 became downregulated in MDA-MB-231 but not in MDA-MB-231R. When miRNAs-449 mimics were added, alone or in combination with doxorubicin, all proteins became downregulated both in sensitive and resistant cell lines; in parallel, protein expression was normally recovered after miRNAs-449 inhibitors transfection, alone or in combination with treatment (Fig. 4C).

### Doxorubicin treatment association with E2F1 in breast cancer samples

**Figure 5:**
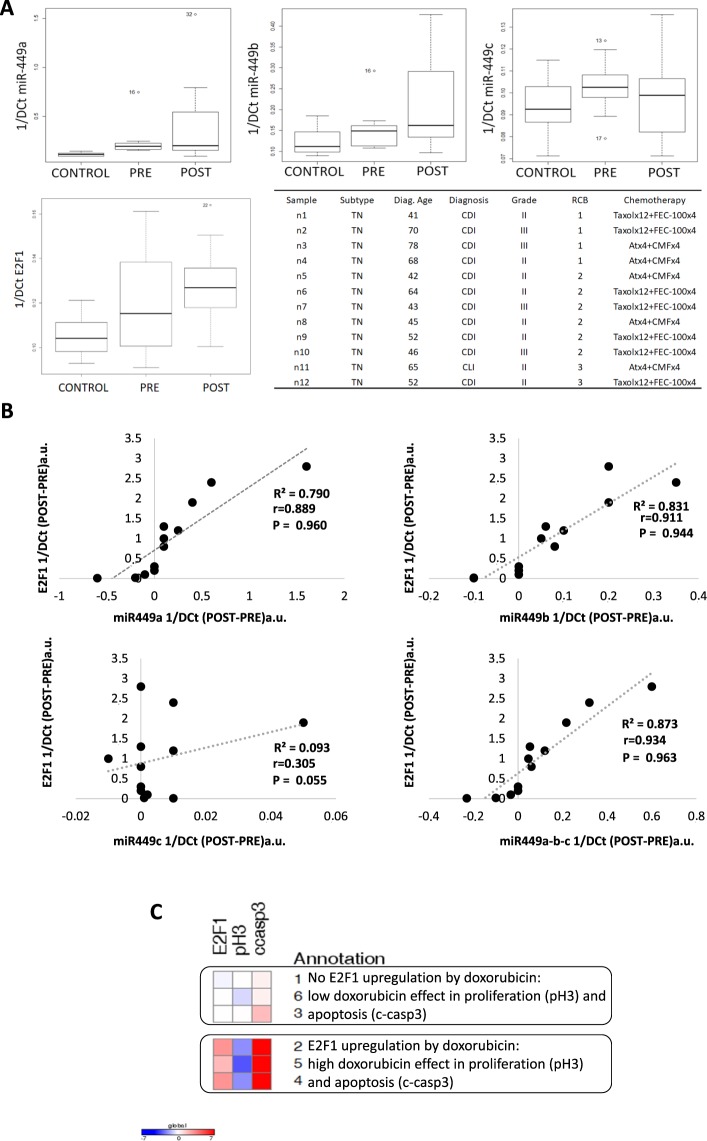
Levels of miR-449 and E2F1 expression in triple negative breast patients. (**A**) Gene expression of twelve paired triple negative breast samples and eight controls was measured by real time PCR for miR-449a, b and c and for *E2F1* in terms of [1/DCt surgery (POST) − 1/DCt biopsy (PRE)]. Summary of the selected patients for the neoadjuvant breast cancer study. TN: triple negative, Age diag.: age at diagnosis, CDI: infiltrating ductal carcinoma, CLI: infiltrating lobular carcinoma, RCB: residual cancer burden; QT observations: neoadjuvant chemotherapy, FEC: (5-Fluoruracil/Epirubicin/Cyclophosphamide), CMF: (cyclophosphamide/methotrexate/5-fluorouracil), Atx: (doxorubicin/paclitaxel). (**B**) Correlation analysis between miR-449 family members and E2F1 expression. The coefficient of determination (R^2^) was determined in order to assess the proportion of the variance in one variable that was predictable from the other. The Pearson (r) and Spearman (P) correlation coefficients were obtained to measure correlation between this two variables (value between +1 and −1, where 1 is total positive linear correlation, 0 is no linear correlation, and −1 is total negative linear correlation). PRE: sample from biopsy/before treatment; POST: sample after surgery/neoadjuvant treatment. (**C**) Immunohistochemical result of *ex vivo* doxorubicin exposition of triple negative fresh specimens staining for E2F2, pH3 (P-Histone H3) and c-casp3 (cleaved-caspase 3). Data is presented as a matrix. Increment of expression (red) and descent of expression (blue).

Expression of miRNA-449 family (miRNA-449a, miRNA-449b and miRNA-449c) was measured in twelve paired samples of breast cancer patients, before and after being treated with neoadjuvant chemotherapy, and in eighth healthy breast samples. The expression level of this miRNAs was higher in the neoadjuvant-treated samples, compared to their biopsied tumour counterparts (Fig. 5A), showing a tendency of increased expression of miRNA-449a (p = 0.073) and miRNA-449b (p = 0.057). Similarly, the *E2F1*gene increased its expression after treatment. In parallel, the correlation between miRNA-449 family members level and *E2F1* gene expression was performed for each paired sample. The coefficient of determination (r^2^), as well as the Pearson (r) and Spearman (P) correlation coefficients between miRNAs-449 levels and *E2F1* expression were calculated. The coefficients between miRNA-449a level and *E2F1* expression obtained were r^2^ = 0.7908, r = 0.889, and P = 0.960. When the levels of miRNA-449b and the expression of *E2F1* were compared, we obtained r^2^ = 0.83, r = 0.911, and P = 0.944. When we compared miR-449c levels and *E2F1* expression, we obtained r^2^ = 0.09, r = 0.3, and P = 0.055. Consistent with above, the comparison between the three miRNA-449 members together was also positively correlated with the expression of *E2F1*, with values of r^2^ = 0.873 r = 0.934, and P = 0.963 (Fig. 5B).

We next assayed whether the effects of doxorubicin observed *in vitro* also occurred in human breast cancer. For that purpose, we analyzed six fresh triple-negative human breast cancer tumors exposed to doxorubicin for 24 h. After fixed and staining with E2F1, p-Histone H3 and c-caspase 3 antibodies, we evaluated the changes in expression for each specimen induced by doxorubicin. The effect of treatment was confirmed in the six cases by the upregulation of P53 in tumor cells. Only three (cases 2, 5, 4) of the specimens showed upregulation of c-caspase 3 and, consequently, apoptosis activation. The caspase cleavage correlated with an increased E2F1 expression induced by doxorubicin exposition. Moreover, more pronounced p-histone H3 downregulation, a marker of proliferation, was observed in same manner. On the contrary, three tumors (cases 1,3, 6) did not show changes E2F1 after treatment, correlating with minor effects on c-caspase 3 and p-histone H3, suggesting a situation of resistance where doxorubicin is not capable of inducing apoptosis nor of producing an increase of E2F1 (Fig. 5C).

### Prognostic and predictive value of miRNA-449 family in breast cancer

**Figure 6:**
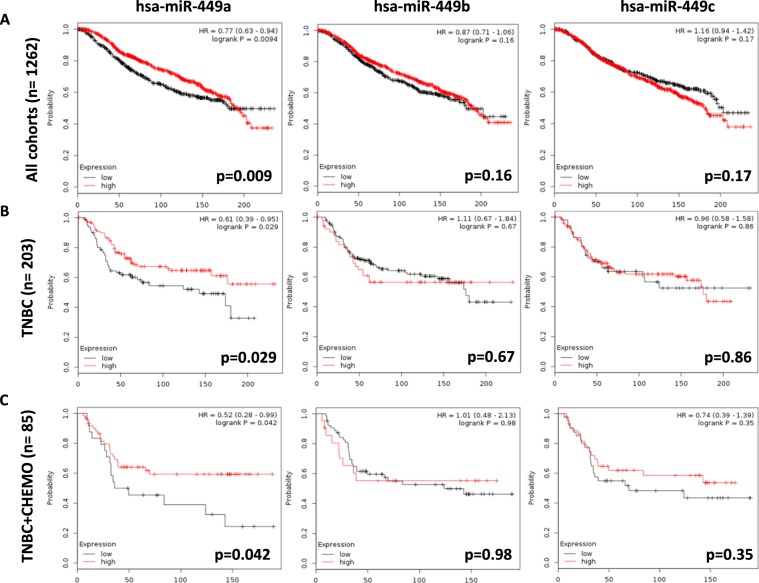
MiR-449 family expression in breast cancer public repositories and its relationship with prognosis. Association between miR-449 family expression and overall survival (OS) in breast cancer patients according to METABRIC study. Kaplan-Meier plots included (**A**) all subtypes of breast cancer patients, (**B**) only triple negative patients, and (**C**) triple negative patients treated with chemotherapy.

In addition, prognostic value of miRNA-449 family expression was obtained (Metabric study-GEO database) through KM-Plot bioinformatic tool. Including all intrinsic subtypes of breast cancer (n = 1262), results showed that high expression of miRNA-449a was significantly associated with good prognosis (log-rank p = 0.0094), meanwhile miRNA-449b (log-rank p = 0.16) and miRNA-449c (log-rank p = 0.17) not. When this cohort was filtered into triple negative patients (TNBC n = 203), miRNA-449a over-expression was again correlated with better overall survival (log-rank p = 0.029). Finally, re-filtering data in triple negative breast patients treated with chemotherapy (n = 85), we obtained that high over-expression of miRNA-449a also correlated with good prognosis (log-rank p = 0.042) (Fig. 6).

## Discussion

Previous work from our group^15^ highlighted the strong upregulation of one particular miRNA family (miRNA-449a, miRNA-449b, and miRNA-449c) after doxorubicin treatment in triple-negative cell lines. Several studies have related this family with tumor suppression^16–20^, proliferation^21,22^, chemo-sensitivity^22^, invasion and metastasis^23–25^ in different types of cancer, and so this present work is focused on these miRNAs at the functional level. Our results showed that the doxorubicin treatment alters the proper functioning of the cell cycle by modulation not only the expression of this miRNA family but also some of its theoretically targeted genes: doxorubicin exposure reduced the expression of the aforementioned genes, except *E2F1*, *E2F3*, and *CDK2*, in MDA-MB-231 and *E2F1* in MDA-MB-468, both triple negative breast cancer cell lines. Overexpression of *E2F1* and *E2F3* can be explained by their role as DNA-damage response genes^26,27^ which require *CDK2* upregulation in order to interact^28^. As described in previous studies^18,21,24^, we also showed that miRNA-449 family influences cell viability, since the transfection of mimics of miRNA-449 reduced cell viability which further decreased in the presence of doxorubicin, and transfection with inhibitors reversed the effect of the drug. These data suggest that overexpression of this family of miRNAs sensitizes breast cancer cells to doxorubicin treatment, bring forward as a possible option for combination therapy. Similarly, miRNAs449 overexpression produced downregulation of genes including *CDK2*, *E2F1*, and *E2F3*, and further decreased of all target genes level in combination with doxorubicin treatment. On the contrary, transfection with miRNAs-449 inhibitors did not alter the expression of their target genes. This data supports the interaction between miRNA-449 and the assayed genes, and the hypothesis that miRNAs-449 represents one mechanism of doxorubicin action and cell cycle deregulation.

The model of acquired resistance to doxorubicin, MDA-MB-231R, did not overexpress miRNA-449-family members after doxorubicin treatment, unlike the wild-type drug-sensitive cell line. Also of relevance is the fact that the expression of genes related to cell-cycle progression did not decrease in this resistant cell line after treatment at the same level as in the sensitive one, highlighting the maintenance of higher levels of *CDK2* expression. In addition, *E2F1* and *E2F3* expression did not increase in response to cell damage. Both *CCNE2* and *CDK2* expression remained high and unchanged after doxorubicin treatment, and it has been described that overexpression and interaction of these two genes is related with tamoxifen-resistance mechanisms in breast cancer^29^. Altogether supports the hypothesis of its role in doxorubicin resistance. MiRNAs-449 overexpression in the doxorubicin-resistant model significantly reduced the expression of the evaluated cell-cycle genes and ultimately, reduced doxorubicin resistance.

**Figure 7:**
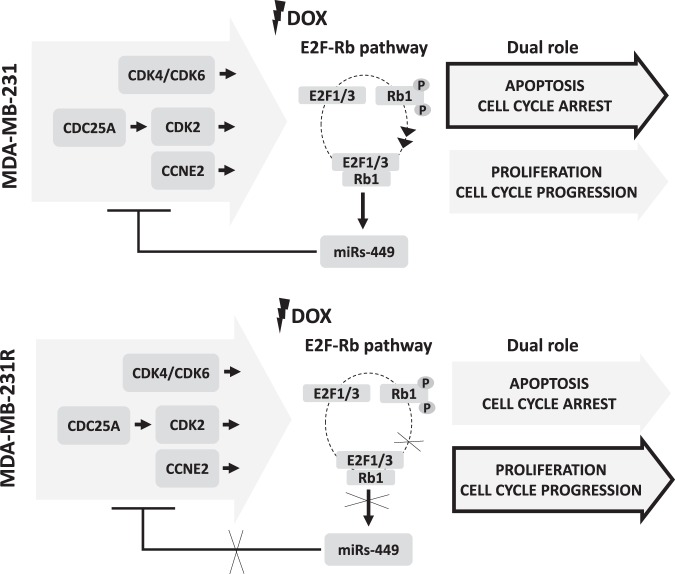
Proposed diagram for miRs-449 regulation in doxorubicin sensitivity and resistance cells. A proposed model for doxorubicin sensitivity/resistance cells by E2F1-miRs-449 circuit coupling the positive and negative feedback loops in cell cycle progression and growth control is shown. In this model, DNA damage produced by doxorubicin, leads into E2F1 directly activation of miR-449 family, which in turn targets and inhibits the expression of factors that belong to the E2F-pRb pathway, including CDK4/6, CCNE2, CDC25A, and E2F1/3. These changes further cause reduced levels of E2F proteins and an increase in hypophosphorylated pRb protein level, which will further reduce E2F activities and thus affect cell cycle progression and growth control. Arrowheads symbolize activation and bars inhibition.

Our experiments demonstrated significant differences in *E2F1*, *E2F3*, *CDK2* and *CCNE2* expression between doxorubicin sensitive and resistant cells. All of these genes are closely related to the RB-E2F pathway which is itself crucial for regulating cell-cycle progression and tumorigenesis. RB-family members interact with the E2F transcription factors forming different types of complexes that either activate or repress transcription^30^. Paradoxically, E2Fs can initiate both cell proliferation and cell death, and so they require tight regulation. The pro-apoptotic role of E2F1 in response to DNA damaging agents is well established, as is its proven involvement in mediating doxorubicin cytotoxicity^31,32^. Doxorubicin treatment increases the Rb1 phosphorylation in the sensitive cell line but not in resistant one where there was not apoptosis induction (Fig. 7). Interestingly, E2F1 strongly and directly upregulates miRNAs-449a/b^33^ and these miRNAs initiate a negative feedback loop that attenuates E2F1 activity by targeting CDK6 and CDC25A^32,33^ through Rb phosphorylation. This feedback provides a safe mechanism to avoid excessive E2F1-induced proliferation. Our results, showing *E2F1* and *E2F3* overexpression induced by doxorubicin in sensitive cells, fit in well with these previous findings. Conversely, *E2F3* downregulation corresponds well with low *E2F1* expression in MDA-MB-231R. Moreover, the forced overexpression of miRNAs-449 produced downregulation of the cell cycle proteins between them E2F1 and E2F3, providing further evidence for this regulatory feedback loop.

On the other hand, CCNE forms an active kinase complex with CDK2 allowing the expression of genes required for entry into S-phase and DNA replication^34–36^. *CDK2* overexpression in MDA-MB-231 sensitive cells after doxorubicin treatment, accompanied by *CCNE2* downregulation, could lead to the promotion of apoptosis because neither of these molecules can form the complex required to promote S-phase entry described above. However, higher expression levels of both *CDK2* and C*CNE2* in treated and non-treated MDA-MB-231R cells, could allow CDK–CCNE complex formation and thus, cell cycle proliferation, even after treatment. Moreover, our analysis *in silico* also confirmed the association between these two proteins. Exposure to doxorubicin increased the percentage of <G_0_-phase cells and increase apoptotic and necrotic population in the sensitive cell line but not in the resistant one. In contrast, miRNAs-449 overexpression resulted in similar cell-cycle alterations in both cell lines. Our results agree with previous studies showing that miRNAs-449a inhibits cell-cycle progression, producing an accumulation in the sub-G_1_ phase and induction of apoptosis in gastric cells^21^. These findings reinforce the idea that these miRNAs could modulate, in part, the response to doxorubicin by regulating their target genes (Figs 3B,C and 4C).

According to these results, we showed a relationship between *E2F1* expression levels and the response to doxorubicin by *ex vivo* treatment of fresh triple negative specimens, since differences between responder and no responder patients in terms of *E2F1* expression were associated with c-casp3 (marker of apoptosis), and inversely correlated with p-histoneH3 (marker of proliferation). These results supported the apoptotic role of E2F factors described in literature^37^. In concordance with this, the patient’s analysis (no-treated versus treated breast tumour paired samples), showed an expression increment of miRNA-449 family, as well as *E2F1* gene expression after chemotherapy, thus suggesting a positive correlation between them and highlighting their role in treatment response. In addition, we have observed a pronounced association between the high expression levels of miRNA-449a and a favourable course of the disease, in terms of greater overall survival, in three different cohorts of breast cancer patients (all molecular subtype, triple negative patients and chemotherapy treated triple negative patients).

Our study has for the first time documented the favourable prognostic significance of miRNA-449a overexpression in patients with breast cancer. We, therefore, propose the miRNA-449a and some of its target genes as possible predictive markers of doxorubicin response in TNBC. In addition, overexpression of this family of miRNAs increases doxorubicin sensitivity, which can be advantageous in terms of using lower drug doses, thus reducing patient side effects. On another hand, the fact that the expression of this family of miRNAs remains unaltered in resistant cells supports the idea that it is implicated in apoptosis and that doxorubicin resistance is produced by cell cycle regulation (Fig. 7). In addition, we had seen evidence for a resistant-cell sensitization mechanism after introducing miRNA-449-family mimics, and this may prove to be important for devising ways to revert the development of resistance at the level of clinical application.

## Materials and Methods

### Cell culture

The MDA-MB-231, MDA-MB-468, and MCF-7 breast cancer cell lines were cultured in a humidified air incubator at 37 °C with 5% CO_2_. MDA-MB-231 and MDA-MB-468 were grown in Dulbecco’s Modified Eagle medium comprising nutrient mixture F-12 (DMEM/F12) with 2.5 mM L-Glutamine and 15 mM HEPES (Gibco), supplemented with 10% FBS and 1% penicillin-streptomycin. MCF-7 cells were grown in DMEM/F12 with 1 g/l D-Glucose, 4 mM L-Glutamine, and 1 mM Pyruvate (Gibco), supplemented with 10% FBS and 1% penicillin-streptomycin. The MDA-MB-231R cell line resistant to doxorubicin was generated by exposing the cells to increasing concentrations of doxorubicin. The MDA-MB-231 cell line was subjected to five cycles of treatment alternating with rest periods until reaching a 70% of cell confluence. The treatment was started with a concentration of 1 uM, and it was doubled in each block of five cycles, until reaching a final concentration of 128 uM. After each treatment cycle, resistance was measured by MTT cell viability assays. Stock solution of 10 mM doxorubicin (Ferrer Farma) and further dilutions were prepared in deionized water as previously described^4,38^. The viability doxorubicin effect on MDA-MB-231 and MDA-MB-231R was evaluated at different concentrations (0.1, 1, 5, 10, and 20 µM), and 5 µM was selected for further experiments (Fig. 2A).

### PCR real time expression analysis

The expression of *CDC25A, CDK2, E2F1, E2F3, SIRT1, BCL-2, GMNN*, and *CCNE2* mRNA and levels of miRs-449 were determined by real-time qPCR. First, 1000 ng RNA was retro-transcribed to cDNA using High-Capacity cDNA Reverse Transcription kit (Applied Biosystems) and TaqMan^®^ MicroRNA Reverse Transcription kit; cDNA was then synthesized at either 25 °C for 10 min and 37 °C for 2 h for mRNA or 16 °C for 30 min, 42 °C for 30 min, and 85 °C for 5 min for miRNA. TaqMan^®^ primers for the *RNU43* housekeeping miRNA as well as for miRNAs-449 were obtained from Applied Biosystems. The transcript levels were detected using a 9700HT Fast Real-Time PCR system (Applied Biosystems); reactions were performed with a TaqMan^®^ Universal Master Mix (Applied Biosystems) and TaqMan^®^ 20 × assay following the manufacturer’s protocol. PCR conditions were: 50 °C for 2 min, 95 °C for 10 min, 40 cycles of 95 °C for 15 sec, and 60 °C for 1 min for gene expression, or 95 °C for 10 min, followed by 40 cycles of 95 °C for 15 sec, and 60 °C for 1 min for miRNA expression. Results were normalized according to the expression of *GAPDH* housekeeping for mRNA or *RNU43* housekeeping for miRNAs. The threshold cycle value (CT) was determined for each measurement and mRNA or miRNA expression was calculated relative to the control using the comparative critical threshold (2−ΔΔCT) method. Each experiment was performed in technical and biological triplicate. The data are represented as the mean plus or minus standard deviation (SD±). The two groups were compared using a Student two-tailed *t*-test; *p*-values less than 0.05 were considered to be statistically significant.

### Analysis of microRNA–gene and protein–protein interactions

The miRNA–gene interactions were computationally predicted and combined using miRWalk v2.0 software (42); it combines miRNA binding sites within the complete gene sequence and also compares the results from 12 different miRNA–gene interaction prediction programs (DIANA-microT v4.0, Diana-microT-CDS, miRanda, miRBridge, miRDB v4.0, miRmap, miRNAMap, PicTar2, PITA, RNA22 v2, RNAhybrid v2.1, and Targetscan v6.2), and miRPath v.3 software^39^. STRING software (http://string-db.org) was used to annotate and analyze each protein–protein interaction with a confidence score indicator, ranking them from 0 to 1, with 1 being the highest possible level of confidence.

### Transient transfection of miRNAs

The cell lines were transfected in six-well plates with either mimic or inhibitor miRNAs (50 nM) for modulating miRNA expression levels, and then exposed to 5 µM of doxorubicin for 24 h. MiRNA mimics are synthetic double-stranded RNAs that act as functional equivalents to endogenous human miRNAs and causes an overexpression of a miRNA of interest. On the other hand, miRNA inhibitors are single-stranded, modified RNAs which specifically inhibit endogenous miRN**A** molecules and causes a down-regulation of miRNA activity. MiRNA-449 mimics and inhibitors (hsa-miRNA-449a mimic (#MC11127), hsa-miRNA-449b-3p mimic (#MC15179), hsa-miRNA-449c-3p mimic (#MC16006), hsa-miRNA-449a inhibitor (#MC11127), hsa-miRNA-449b-3p inhibitor (#MC15179), and hsa-miRNA-449c-3p inhibitor (#MC16006)) were purchased from Ambion (Austin, TX). CyTM3-labeled pre-miRNA (#AM17120; Ambion) was used as a negative transfection control. The reactions were performed with the TransIT-X2 Dynamic Delivery System reagent (Mirus, Wisconsin, USA), following the manufacturer’s instructions.

### MTT cell viability assay

Cell viability was measured using an MTT-based Cell Growth Determination kit (# GDC1; Sigma, St Louis MO, USA). The MTT solution was added to each well in sterile conditions (the final concentration was 10% of total volume) and the plates were incubated for 4 h at 37 °C. The formazan crystals formed and were dissolved in solubilization solution (1:1); the purple formazan crystals were formed from yellow MTT by succinate dehydrogenase in viable cells. The absorbance of the dissolved formazan product was measured at a 570 nm background corrected to 690 nm using a microplate reader. Each experiment was performed in triplicate and statistically significant differences were evaluated using a Student *t*-test.

### Flow cytometry assays

The percentage of apoptotic cells 24 h after doxorubicin treatment was measured by flow cytometry (Becton Dickinson, NJ, USA). Cell labeling was performed with 0.5 mg/ml of FITC Annexin V-conjugated fluorochrome and 0.5 mg/ml o of DAPI and incubated for 15 min in darkness at room temperature using an FITC Annexin V Apoptosis Detection Kit I (BD Pharmingen^™^). For cell cycle analysis, cells were kept on ice prior to fixation. After detaching the cells with trypsin, 2 × 10^6^ cells were added per cytometer tube. Cells were fixed with 1 ml cold 80% ethanol and incubated for 2 h with ethanol at −20 °C. Finally, the cells were re-suspended with 1 ml DAPI/TX-100 solution (Sigma, St. Louis MO, USA), and incubated for 30 min at room temperature before flow-cytometry analysis. The samples were filtered prior to acquisition in order to eliminate any cell aggregates. The wavelengths of excitation and emission for DAPI were 405 nm and 450 nm respectively.

### Western immunoblotting

Cells were seeded and then exposed to treatment and/or transfection experiments. After double wash with cold PBS, the monolayers were scraped into 1 ml of Pierce RIPA buffer (Thermo #89900). The lysates were transferred to a clean microfuge tube, placed on ice for 15 min, sonicated 30 sec with 50% pulse, and then centrifuged for 10 min at 14,000 rpm. The supernatant was transferred to a clean microfuge tube, and the protein concentration was determined. Protein extracts (15 μg) were boiled in Laemmli buffer and resolved on a 12% SDS-polyacrylamide gel, before transfer onto a nitrocellulose membrane. Membranes were blocked in 5% BSA for 1 hour and then incubated with antibodies to E2F1 (SantaCruz, #sc-251), E2F3 (SantaCruz, #sc-69683), CDK2 (SantaCruz, #sc-6248), CCNE2 (Abcam, #40890), CDK4 (Abcam#137675), CDK6 (Abcam#151247), CDC25A (Thermofisher #MA5-13794), pRb1-Ser795 (Thermofisher#PA5-38146), βACTIN (Cell Signaling#12620 S), GAPDH (Invitrogen #MA5-15738), overnight at 4 °C. The membranes were subsequently washed and then incubated for 1 hour with an anti-mouse or anti-rabbit IgG horseradish peroxidase-linked secondary antibody (Cell signaling, #7076 and #7074). The membranes were then washed and briefly incubated using with an Amersham ECL Western Blotting detection reagent (GE Healthcare, #RPN2209). All images were analysed as TIFF files with Image J k 1.45 for windows to build the figures. Graphs of signal intensity were obtained through band densitometry.

### Patient samples

#### *Ex vivo* human breast cancer model

Six fresh surgical specimens of patients diagnosed with triple negative breast cancer were obtained to add *ex* vivo doxorubicin and to assess molecular effects. The sample were processed in sterile conditions immediately after surgical resection. Incubation with doxorubicin (2ug/ml) was performed in 24-well plates at 37 °C in a constant atmosphere of 5% CO2. After 24 hours, the specimens were fixed in 10% neutral-buffered formalin for 16 hours at room temperature and embedded in paraffin under vacuum conditions. The immunostaining was performed on 3um tissue sections. After deparaffinization, heat antigen retrieval was performed in pH9 EDTA-based buffer (Dako). Endogenous peroxidase was blocked and slides were incubated with primary antibodies: mouse monoclonal E2F1 (SantaCruz, clone HK95, #sc-251), rabbit polyclonal phosphorylated (p)-Histone H3 Ser10 (Cell Signaling, CST, #9701) and rabbit polyclonal cleaved (c)-caspase 3 Asp175 (CST, #9661) for 60 min, followed of anti-Ig horseradish peroxidase-conjugated polymer (Flex + Dako) and visualized with 3,3′-diaminobenzidine.

#### Neoadjuvant breast cancer analysis

Twenty-four formalin-fixed paraffin-embedded (FFPE) samples of twelve triple negative breast cancer patients (Supplementary Table 2) were selected to analyze the expression changes of miRNA-449a, miRNA-449b, miRNA-449c and *E2F1* gene, between untreated tumor samples (biopsy) and anthracyclines neoadjuvant treated samples (surgery). In addition, eighth healthy breast samples were analyzed as control group. Genetic material was isolated from FFPE tissue blocks using the RecoverAll Total Nucleic Acid Kit (Ambion). RNA was extracted from manually microdissected areas of 4 tissue sections (10 μm thick) on glass slides selected by a pathologist for each relevant FFPE tissue block. For standard mRNA/miRNA analysis, 1 µg of total RNA (RNA concentration measured using the NanoDrop® Spectrophotometer) was reverse transcribed with random primers using the High Capacity cDNA Reverse Transcription Kit (Applied Biosystems) and 5 ng of cDNA from FFPE tissue, was then analyzed per reaction by PCR. In case of pre-amplification, 25 ng of total RNA from FFPE tissue blocks were reverse transcribed, pre-amplified for 14 cycles using the 2X TaqMan PreAmp Master Mix (Applied Biosystems) according to manufacturer’s instructions, and diluted 1:5 prior to PCR analysis. Quantitative PCR analysis was performed as mentioned above. Ethical approval for the study was obtained from the Research Ethics Committee of the Hospital Clínico Universitario de Valencia (Spain). All patients signed written informed consent for study enrolment.

#### Survival analysis

Kaplan-Meier plotter (KM plotter ©) tool was used to evaluate the predictive/prognostic value of miRNA-449 family members on patient survival. By entering the interesting miRNAs to the blank of the website, breast cancer patients from GEO datasets (Metabric study) were divided into two groups according to the expression level of each miRNA with auto-selected best cut-off, and statistically analyzed the survival rate. The hazard ratio (HR) with 95% confidence intervals and log rank p-value were calculated and showed. The obtained results were used to identify the distinct prognostic values of miRNA-449 family on breast cancer^40^.

The authors declare that all experiments were performed in accordance with relevant guidelines and regulations.

## Supplementary information

Supplementary material
