# Supplementary material for: The miRNA-449 family mediates doxorubicin resistance in triple-negative breast cancer by regulating cell cycle factors
Source: Sci Rep. 2019 Mar 29;9:5316. doi: 10.1038/s41598-019-41472-y (PMC6441107; doi:10.1038/s41598-019-41472-y)
Supplement: Supplementary file 1 — Supplementary material [file 41598_2019_41472_MOESM1_ESM.pdf]

Title:The miRNA-449 family mediates doxorubicin resistance in triple-negative breast cancer by regulating cell cycle factors

Authors: Eduardo Tormo, Sandra Ballester, Anna Adam-Artigues, Octavio Burgués, Elisa Alonso, Begoña Bermejo, Silvia Menéndez, Sandra Zazo, Juan Madoz-Gúrpide, Ana Rovira, Joan Albanell, Federico Rojo, Ana Lluch and Pilar Eroles.

A

Name	miRBase name	Sequence	MDA-MB-231		MDA-MB-468		MCF-7	
			Fold change	*p*-value	Fold change	*p*-value	Fold change	*p*-value
hsa-miR-449a	hsa-miR-449a	UGGCAGUGUAUUGUUAGCUGGU	2.38504	0.00362624	2.27526	0.00519606	1.05291	0.834354
hsa-miR-449b	hsa-miR-449b-5p	AGGCAGUGUAUUGUUAGCUGGC	2.83171	8.59318E-05	2.04142	0.00185811	1.28812	0.184337
hsa-miR-449b*	hsa-miR-449b-3p	CAGCCACAACUACCCUGCCACU	1.56305	0.0186991	1.57722	0.0168919	-1.14728	0.419531
hsa-miR-449c	hsa-miR-449c-5p	UAGGCAGUGUAUUGCUAGCGGCUGU	4.17516	0.000007605	3.30549	4.2991E-05	1.55477	0.0398724

B

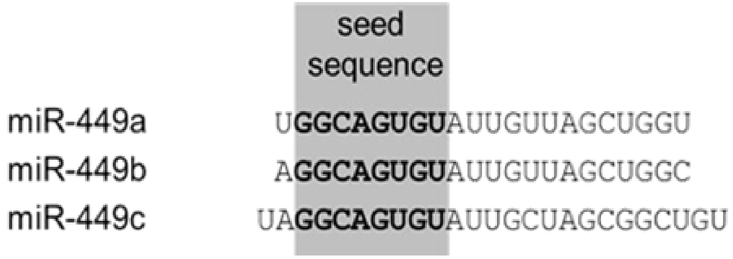

C

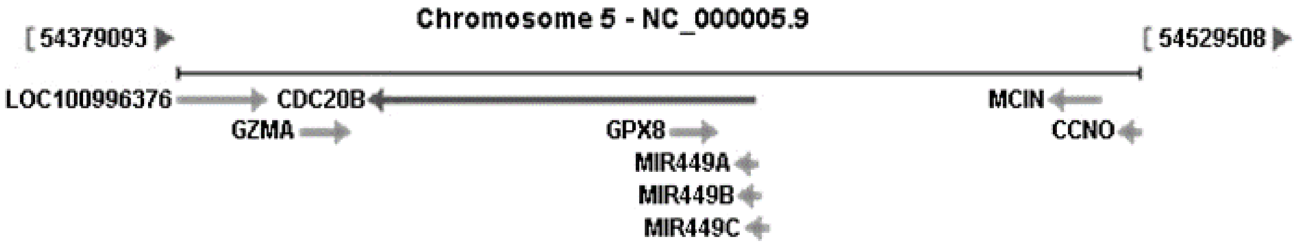

Supplementary Figure 1. MiRNA-449-family expression in breast cancer cell lines. A) Changes in miRNA-449-family expression after doxorubicin treatment in three breast cancer cell lines (MDA-MB-231, MDA-MB-468 and MCF-7); data obtained in our previously published study (15). B) Alignment of the mature miR-449 family member sequences; modified from Lizé et al. (36), and C) Genomic localization of miR-449 family members on chromosome 5q11.2.

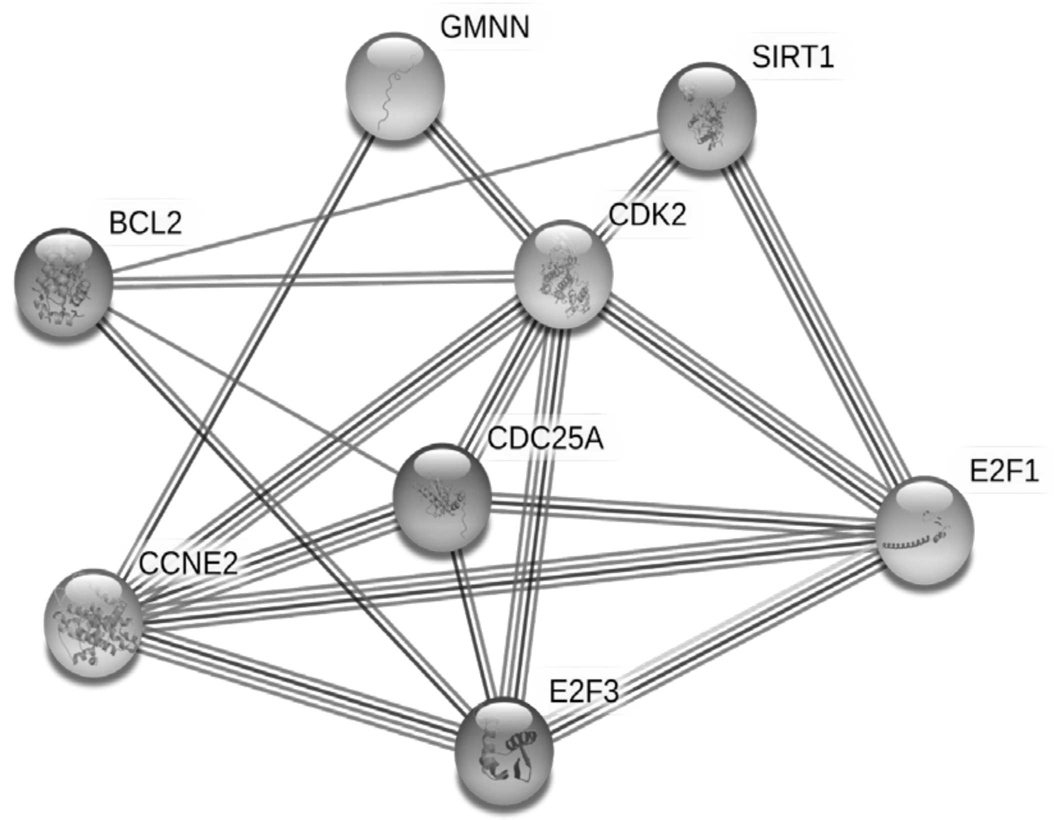

node1	node2	node1 accession	node2 accession	score
BCL2	E2F3	ENSP00000329623	ENSP00000262904	0.404
BCL2	CDC25A	ENSP00000329623	ENSP00000303706	0.449
CCNE2	GMNN	ENSP00000309181	ENSP00000230056	0.488
BCL2	SIRT1	ENSP00000329623	ENSP00000212015	0.503
CDC25A	E2F3	ENSP00000303706	ENSP00000262904	0.504
CDK2	SIRT1	ENSP00000266970	ENSP00000212015	0.512
CDK2	GMNN	ENSP00000266970	ENSP00000230056	0.861
BCL2	CDK2	ENSP00000329623	ENSP00000266970	0.894
E2F1	E2F3	ENSP00000345571	ENSP00000262904	0.907
CCNE2	E2F1	ENSP00000309181	ENSP00000345571	0.942
CDC25A	E2F1	ENSP00000303706	ENSP00000345571	0.945
E2F1	SIRT1	ENSP00000345571	ENSP00000212015	0.947
CCNE2	CDC25A	ENSP00000309181	ENSP00000303706	0.969
CCNE2	E2F3	ENSP00000309181	ENSP00000262904	0.974
CDK2	E2F3	ENSP00000266970	ENSP00000262904	0.975
CDK2	E2F1	ENSP00000266970	ENSP00000345571	0.983
CDC25A	CDK2	ENSP00000303706	ENSP00000266970	0.998
CCNE2	CDK2	ENSP00000309181	ENSP00000266970	0.999

Supplementary Figure 2. Study of the interactions between the products of the different miRNA-449-family target genes. Source: STRING software. The network view summarizes the network of predicted associations for a particular group of proteins: the network nodes are proteins; the edges represent the predicted functional associations and shows additional information about the prediction such as, binding, activation, etc. The table represents the confidence score, the approximate probability that a predicted link exists between two enzymes in the same metabolic map in the KEGG database. The confidence score limits are as follows: low confidence = 0 ≥ 0.15, medium confidence = 0.15 ≥ 0.4, high confidence = 0.4 ≥ 0.7, highest confidence = 0.9 ≥ 1.0.

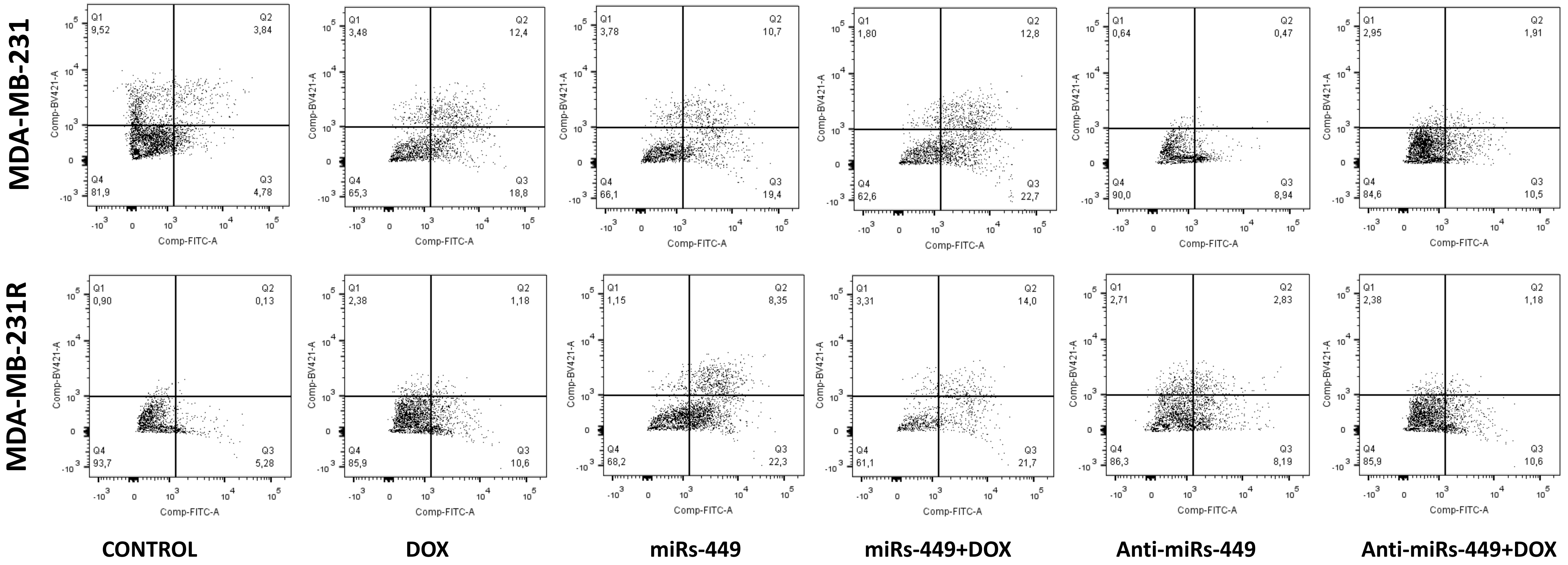

Supplementary Figure 3. Flow cytometry apoptosis study in MDA-MB-231 and MDA-MB-231R cells treated with doxorubicin and transfected with miRNA-449- family mimics and inhibitors and treated with doxorubicin. Percentage of living cells (Q4), early apoptotic cells (Q3), late apoptotic cells (Q2) and necrotic cells (Q1). Cells were marked with Annexin V (Y axis)/DAPI (X axis).

Gene	Mirwalk	MicroT4	miRanda	mirbridge	miRDB	miRNAMap	Pictar2	PITA	RNA22	RNAhybrid	Targetscan	SUM
E2F3	1	1	1	0	1	1	1	1	1	1	1	10
SIRT1	1	1	1	0	0	1	1	1	1	1	1	9
CCNE2	1	1	1	0	1	1	1	1	0	1	1	9
CDC25A	1	1	0	0	0	1	0	0	1	1	1	6
GMNN	0	1	0	0	0	1	0	0	0	1	0	3
CDK2	0	1	0	0	0	0	0	0	0	1	0	2
BCL2	0	0	0	0	0	1	0	0	0	1	0	2
E2F1	0	0	0	0	0	0	0	1	0	1	0	2

Supplementary Table 1. Bioinformatic analysis of miR-449-family target genes using miRWalk v2.0 software showing genes that may be affected by doxorubicin treatment. SUM: Sum of the number of bioinformatic programs (from a total of 12) that predicted each miRNA–gene interaction.

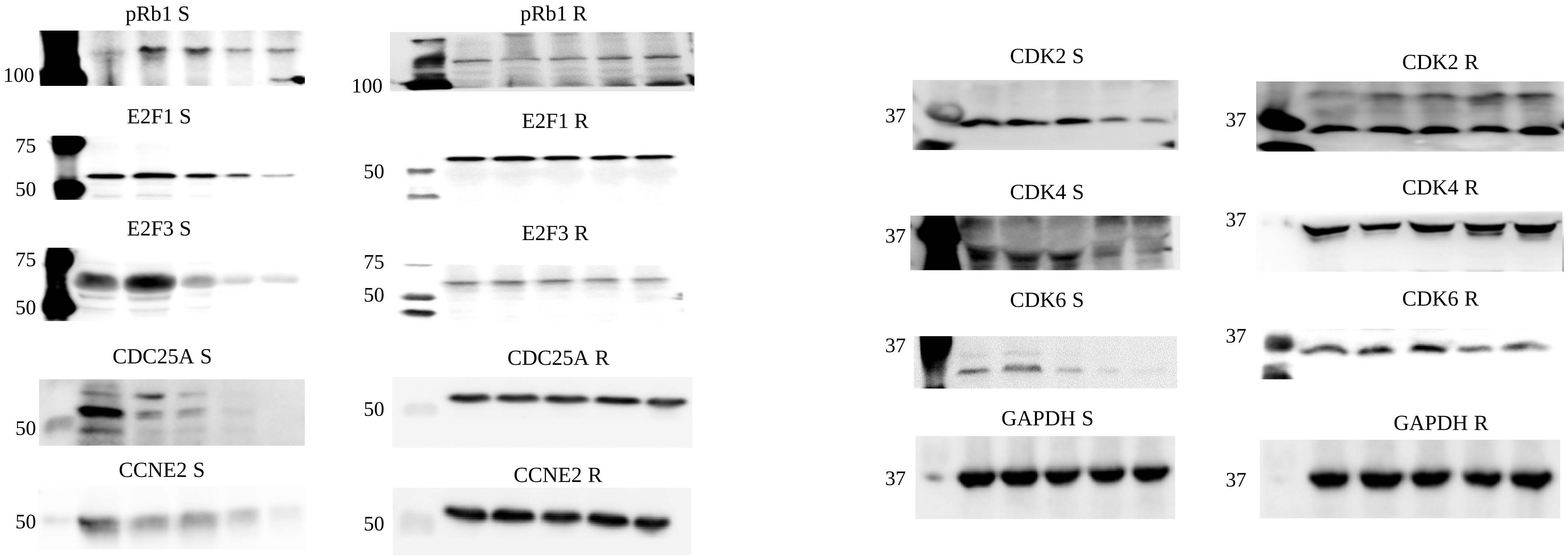

Supplementary Figure 4. Original western blott images from figure 3C.Developing membranes were analyzed with ImageQuant LAS 4000 technology from GE healthcare lifesiences. Membranes were developed under “Precision” Exposure Type, “Manual” Exposure Time, “Standard” Sensitivity/Resolution, with Digitization images. In each case all proteins coincided with their molecular weight markers: E2F1 (~60KDa), E2F3 (~60KDa), CDK2 (~34KDa), pRb1(~100KDa), CDK4 (~34KDa), CDK6 (~34KDa), CDC25A (~50KDa), CCNE2 (~50KDa), GAPDH (~34KDa). All images were analyzed as TIFF files with Image J k 1.45 for windows to build the figures, adjusting Brightness/Contrast. On paper final image construction, weight markers were removed, and all bands were adjusted with similar width and height, as well as background color.

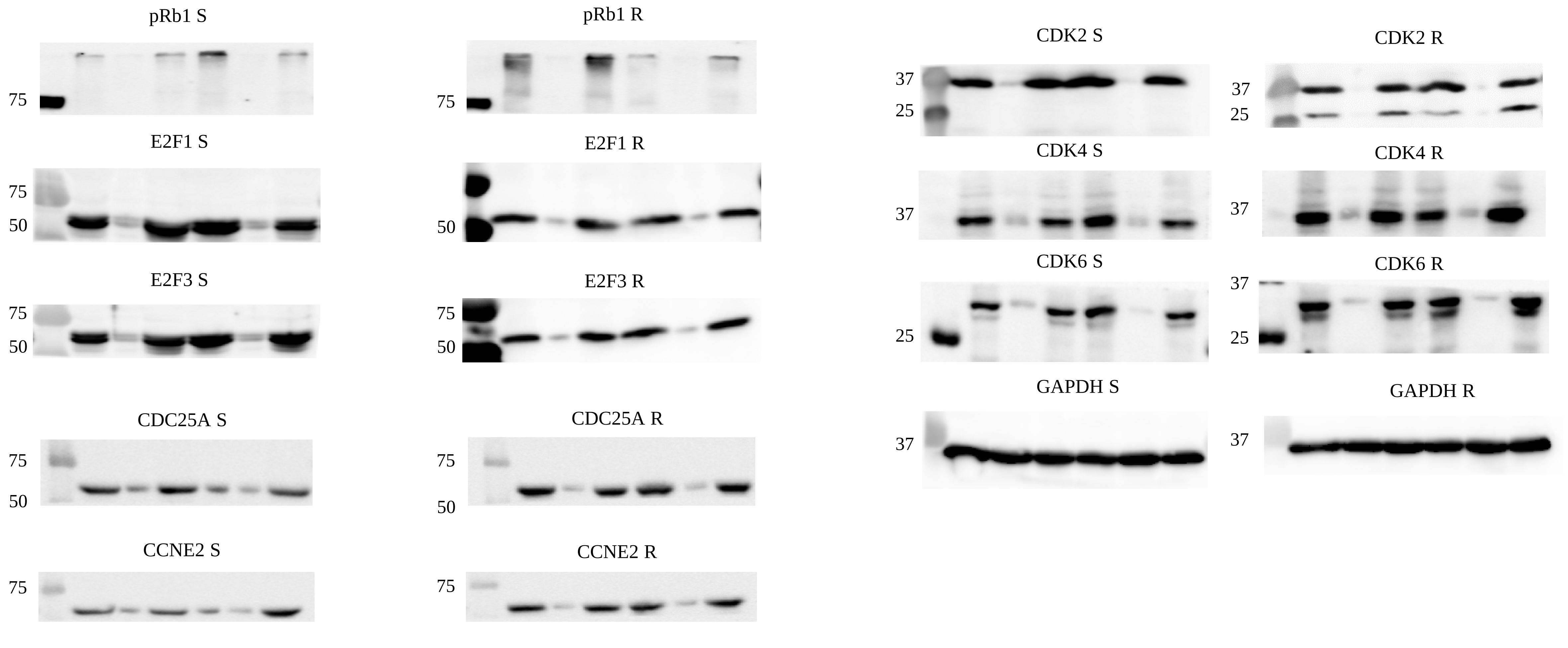

Supplementary Figure 5. Original western blott images from figure 4C.Developing membranes were analyzed with ImageQuant LAS 4000 technology from GE healthcare lifesiences. Membranes were developed under “Precision” Exposure Type, “Manual” Exposure Time, “Standard” Sensitivity/Resolution, with Digitization images. In each case all proteins coincided with their molecular weight markers: E2F1 (~60KDa), E2F3 (~60KDa), CDK2 (~34KDa), pRb1(~100KDa), CDK4 (~34KDa), CDK6 (~34KDa), CDC25A (~50KDa), CCNE2 (~50KDa), GAPDH (~34KDa). All images were analyzed as TIFF files with Image J k 1.45 for windows to build the figures, adjusting Brightness/Contrast. On paper final image construction, weight markers were removed, and all bands were adjusted with similar width and height, as well as background color.
